# A review of the clinical efficacy of FDA-approved antibody‒drug conjugates in human cancers

**DOI:** 10.1186/s12943-024-01963-7

**Published:** 2024-03-23

**Authors:** Kaifeng Liu, Meijia Li, Yudong Li, Yutong Li, Zixin Chen, Yiqi Tang, Meitian Yang, Guoquan Deng, Hongwei Liu

**Affiliations:** 1 Laboratory of Urology, Affiliated Hospital of Guangdong Medical University, Zhanjiang, 524001 China; 2 The First Clinical College, Guangdong Medical University, Zhanjiang, 524023 China

**Keywords:** Antibody‒drug conjugates, Cancer therapy, Targeted drugs, FDA‒approved, Clinical efficacy

## Abstract

While strategies such as chemotherapy and immunotherapy have become the first-line standard therapies for patients with advanced or metastatic cancer, acquired resistance is still inevitable in most cases. The introduction of antibody‒drug conjugates (ADCs) provides a novel alternative. ADCs are a new class of anticancer drugs comprising the coupling of antitumor mAbs with cytotoxic drugs. Compared with chemotherapeutic drugs, ADCs have the advantages of good tolerance, accurate target recognition, and small effects on noncancerous cells. ADCs occupy an increasingly important position in the therapeutic field. Currently, there are 13 Food and Drug Administration (FDA)‒approved ADCs and more than 100 ADC drugs at different stages of clinical trials. This review briefly describes the efficacy and safety of FDA-approved ADCs, and discusses the related problems and challenges to provide a reference for clinical work.

## Introduction

Cancer has emerged as the second-largest global threat to people’s health, causing ∼ 10 million deaths in 2020 [1]. Traditional antitumor therapies, such as chemotherapy and radiotherapy, exhibit numerous drawbacks [2]. To address this challenge, scientists have identified a novel class of cancer therapy drugs known as antibody‒drug conjugates (ADCs), which offer enhanced safety and efficacy [3]. The U.S. Food and Drug Administration (FDA) marked the initiation of cancer-targeted ADC therapy in 2000 by approving the ADC drug Mylotarg for treating patients with acute myeloid leukemia (AML) meeting specific criteria (i.e., first relapse, over 60 years old, CD33-positive, and unsuitable for cytotoxic chemotherapy) [4]. Since the introduction of the first ADC, 13 ADCs have secured FDA approval, and over 100 ADCs are currently undergoing various stages of clinical research [5].

**Fig. 1:**
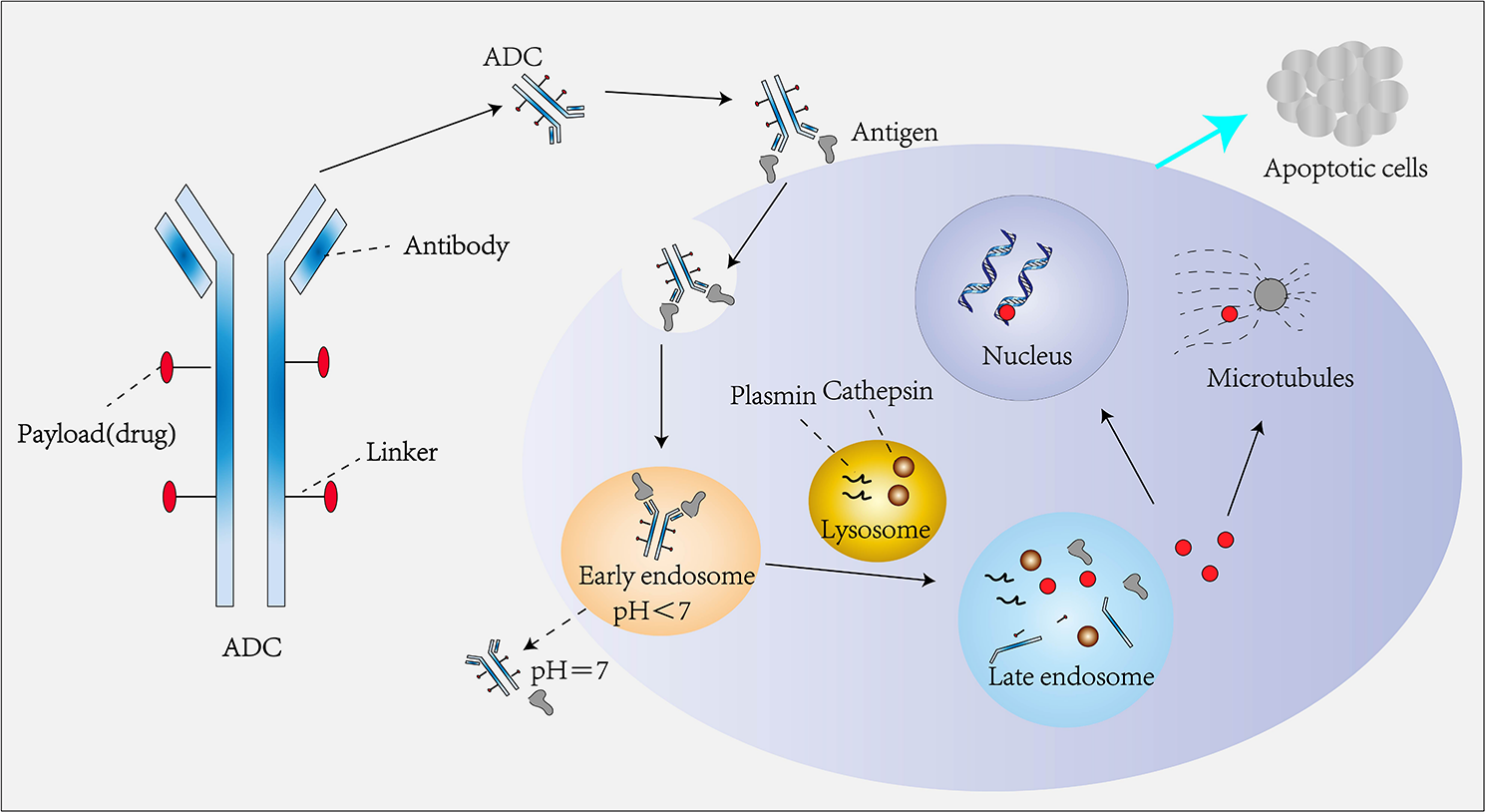
Structure and mechanism of action of ADCs

An ADC comprises three primary components: human-derived monoclonal antibodies (mAbs), a linker, and a cytotoxic drug [6]. The mAb within the ADC recognizes antigens on the target cell’s membrane, facilitating its entry into the cell through endocytosis. In most cases, the mAb is translocated to early endosomes and subsequently to lysosomes. The acidic environment and protein hydrolases within these compartments result in ADC degradation, releasing the cytotoxic drug into the cytoplasm. The released cytotoxic drug then binds to DNA or microtubule proteins, causing cell cycle arrest and eventual apoptosis [7] (Fig. 1).

As a novel and promising therapeutic agent, ADCs have a wide range of potential applications. This paper will provide a brief review of the efficacy and safety of FDA-approved ADCs (Table 1), offering references for both clinical application and scientific research.

## ADCs and their role in cancer therapy

### Gemtuzumab ozogamicin (GO)

GO, the pioneering ADC drug developed by Pfizer, holds the distinction of being the first ADC to receive global market approval. Comprising a humanized mAb targeting CD33 and a cytotoxic N-acetyl-γ-calicheamicin connected via a cleavable hydrazone linker, GO operates on a therapeutic principle designed for patients with AML [8]. The mechanism involves GO binding to the CD33 antigen, forming the GO–CD33 complex, which is then internalized into AML primary cells [9]. Following positive outcomes from three early clinical trials, the FDA granted approval of GO in 2000, specifically for the treatment of patients with CD33-positive AML aged over 60 who were ineligible for cytotoxic chemotherapy [10].

However, safety concerns surfaced during the Southwest Oncology Group (SWOG) S0106 study, designed to assess the efficacy of GO across all cytogenetic risk groups in adult patients below 60 years of age with AML. The study revealed a higher fatal induction toxicity rate in the GO + cytarabinealone group compared to the cytarabinealone group (5.5% vs. 1.4%) [11]. Consequently, Pfizer withdrew the product from the market in June 2010. The safety and therapeutic efficacy of GO were reevaluated at a lower dose (3 mg/m²) in combination with chemotherapy in the Acute Leukemia French Association (ALFA)-0701 phase III clinical trial. The study indicated varying incidences of grade ≥ 3 adverse events (AEs) in the GO combined with chemotherapy and chemotherapy alone groups, including veno-occlusive liver disease (2% vs. 0%), hemorrhage (18% vs. 9%), and infections (47% vs. 39%), respectively [12].

Additional trials, namely MyloFrance-1 and AML-19, were conducted to assess the safety and efficacy of GO [4, 13]. The MyloFrance-1 study demonstrated significant toxicities associated with GO treatment, such as myelosuppression, infusion reactions, infections, bleeding, and hepatotoxicity. However, the data suggested that the anticipated clinical benefits for patients with CD33-positive relapsed/refractory AML outweighed safety concerns when treated with 3 mg/m² GO on days 1, 4, and 7 [4]. In the AML-19 study focusing on overall survival (OS), results indicated a promising improvement in OS for elderly patients with AML unsuitable for intensive chemotherapy compared to best supportive care. The toxicity was manageable, with no additional adverse effects observed [13]. Based on these studies, the FDA granted approval of GO in 2017 [14]. Subsequently, in pediatric AML, the Children’s Oncology Group’s AAML0531 trial demonstrated improved prognosis for pediatric patients treated with GO [15].

The final efficacy and safety update from the open-label, phase III ALFA-0701 trial revealed that the addition of GO to standard chemotherapy significantly extended event-free survival (EFS) in patients with newly diagnosed *de novo* AML [16]. In a randomized, open-label, multicenter phase III trial (AMLSG 09–09), the primary investigation focused on the efficacy of intensive chemotherapy with or without GO in patients with NPM1 mutant AML. The results demonstrated a significant reduction in the cumulative relapse rate when GO was combined with chemotherapy. AEs (grade ≥ 3) and their incidence in the GO combined with chemotherapy and chemotherapy alone groups included febrile neutropenia (47% vs. 41%), thrombocytopenia (90% vs. 90%), pneumonia (25% vs. 22%), and sepsis (29% vs. 25%), respectively [17].

A retrospective analysis gathered data on 35 children with refractory or relapsed AML treated with GO in Poland from 2008 to 2022. Outcomes indicated that 18 children achieved complete response (CR), 14 did not respond to treatment, and 3 progressed. Among the 18 children with CR after GO treatment, allogeneic hematopoietic stem cell transplantation was performed. The 5-year OS for the entire cohort post-GO treatment was 37.1% ± 8.7%. Patients with strong CD33 expression (more than 50% positive cells) demonstrated a trend towards better outcomes compared to those with low CD33 expression. Common AEs included bone marrow aplasia, unexplained fever, infections, and elevated liver enzymes [18].

In the UK NCRI AML18 trial, investigators explored the benefits of fractionated versus single-dose GO in elderly patients with AML. Results indicated that a fractionated regimen was more effective than a single dose in clearing leukemia in older individuals without adverse genetic risk [19]. In a phase IV study evaluating the QT interval, pharmacokinetics, and safety after fractionated GO administration in patients with relapsed/refractory CD33-positive AML, findings suggested that a fractionated GO dosing regimen did not pose a clinically significant safety risk for QT interval prolongation. Treatment-emergent adverse events (TEAEs) were consistent with the previously reported safety profile of GO [20].

Evidence has shown that GO, when combined with standard induction chemotherapy, enhances the prognosis for newly diagnosed intermediate cytogenetic risk AML [21]. The use of GO in combination with fludarabine, cytarabine, granulocyte colony-stimulating factor, and idarubicin has demonstrated improved EFS in young patients newly diagnosed with AML, and enhanced OS in patients with NPM1 and FLT3 mutations [22]. Collectively, these findings suggest that GO, whether administered as a standalone agent or in combination, slows disease progression and is deemd safe, efficacious, and feasible in patients with CD33-positive AML at their initial diagnosis.

### Brentuximab vedotin (BV)

BV, initially developed by Seagen (formerly Seattle Genetics) and later co-developed with Takeda, stands as the second approved ADC drug. It comprises brentuximab, a chimeric IgG1 mAb targeting CD30, a maleimide linker moiety (a cleavable dipeptide linker, mc–VC–PABC), and monomethyl auristatin E (MMAE). BV specifically targets the CD30 antigen expressed in Hodgkin lymphoma (HL) and anaplastic large cell lymphoma (ALCL) [23].

In a phase I study evaluating the efficacy and safety of BV for the treatment of HL and ALCL, 45 patients with relapsed/refractory CD30-positive hematological malignancies received BV at doses ranging from 0.1 to 3.6 mg/kg of body weight every 3 weeks. Results indicated objective response in 50% of cases, with a median duration of response (DOR) lasting at least 9.7 months. Most AEs were of grade 1 and 2 severity, with the common ones including fatigue, fever, diarrhea, nausea, neutropenia, among others [24]. In a phase II trial, BV demonstrated effectiveness in 75% and 87% of patients with HL (102 patients) and ALCL (30 patients), respectively [25].

A phase III study exploring BV in the treatment of cutaneous T-cell lymphomas revealed significant improvement in mycosis fungoides or primary cutaneous ALCL. Moreover, BV demonstrated the ability to alleviate itch and pain caused by lymphoma without negatively impacting the patients’ quality of life (QoL) [26]. In 2018, the FDA approved BV in combination with CHP (i.e., cyclophosphamide, doxorubicin, and prednisone) for treating adult patients with previously untreated systemic ALCL or other CD30-expressing peripheral T-cell lymphomas (PTCL), including angioimmunoblastic T-cell lymphoma and PTCL not otherwise specified [27].

In a multicenter real-world study conducted between 2020 and 2022, researchers enrolled 104 patients with lymphoma receiving BV for the first time. The results demonstrated an objective response rate (ORR) of 64.5%, with 6-month progression-free survival (PFS) and OS rates reaching 77.2% and 90.1%, respectively. The 12-month PFS and OS rates were reported at 77.2% and 79.9%, respectively. The most prevalent AEs were hematological disorders, particularly neutropenia [28].

In an open-label, single-arm, multicenter phase I/II trial, 41 patients with HIV-related HL received BV in combination with doxorubicin, vinblastine, and dacarbazine. Results indicated that all 37 patients who completed treatment achieved CR. The 2-year PFS was 87%, and the OS rate was 92%. The most common grade 3 or worse AEs included peripheral sensory neuropathy (10%), neutropenia (44%), and febrile neutropenia (12%) [29].

### Trastuzumab emtansine (T-DM1)

T-DM1 is an ADC drug formed by linking the HER2-targeting drug trastuzumab with emtansine (also known as DM1) via a thioether linker. The targeting action of trastuzumab selectively transports the highly active cytotoxic small molecule drug DM1 into tumor cells with HER2 overexpression, releasing the drug through endocytosis. This mechanism not only significantly reduces toxicity and side effects but also enhances the targeting role [30].

The international multicenter phase III clinical trial, EMILIA, conducted by Verma et al., affirmed the clinical role of T-DM1 in HER2-positive advanced breast cancer. In patients with HER2-positive metastases or advanced breast cancer treated with trastuzumab and paclitaxel, T-DM1 demonstrated enhanced treatment efficacy, a higher safety profile, and fewer adverse effects [31]. In the TDM4450g study, T-DM1 showed generally favorable tolerance in patients with HER2-positive metastatic breast cancer. TEAEs with an incidence rate of > 40% included fatigue (49.3%), nausea (49.3%), an increase in serum aspartate aminotransferase (43.5%), pyrexia (40.6%), and headache (40.6%) in the T-DM1 group [32].

The TH3RESA study validated the effectiveness of T-DM1 in breast cancer patients who progressed after second-line and above treatment. Results indicated a significantly improved median PFS in the T-DM1 group, along with a prolonged median OS and a lower proportion of ≥ 3 adverse reactions compared to the control group [33]. A real-world study presented at the 2019 European Society for Medical Oncology (ESMO) congress from a US database confirmed the benefit of T-DM1 in patients who had failed dual-target therapy with trastuzumab and pertuzumab [34]. The NCCN Breast Cancer Guidelines designate T-DM1 as the preferred second-line treatment for HER2-positive advanced breast cancer [35].

In adjuvant therapy for residual invasive HER2-positive early breast cancer, T-DM1 plays a crucial role. The KATHERINE study indicated that the T-DM1 group exhibited an improved 3-year disease-free survival (DFS) rate and a significantly reduced risk of recurrence or death. This study establishes T-DM1 as the new standard treatment for patients with residual lesions after neoadjuvant therapy for HER2-positive breast cancer [36].

In the ATEMPT trial, the objective was to assess whether T-DM1 treatment resulted in lower toxicity compared to paclitaxel plus trastuzumab, while still achieving clinically acceptable invasive DFS in patients with stage I HER2-positive breast cancer. The study revealed that the 3-year invasive DFS for T-DM1 reached 97.8%, and patients treated with T-DM1 experienced less neuropathy and alopecia than those treated with paclitaxel plus trastuzumab [37].

In the WSG-ADAPT-TP phase II trial involving 375 hormone receptor-positive or HER2-positive patients, randomization into three groups (T-DM1, T-DM1 + endocrine therapy, trastuzumab + endocrine therapy) resulted in similar 5-year invasive DFS rates (88.9%, 85.3%, and 84.6%, respectively) and OS rates (97.2%, 96.4%, and 96.3%, respectively) [38].

A phase I trial enrolled 12 patients with HER2-positive breast cancer and brain metastases, investigating the combination of T-DM1 and metronomic temozolomide. The study indicated low-grade toxicity and potential activity in the secondary prevention of HER2-positive brain metastases. Grade 3 or 4 AEs included thrombocytopenia, neutropenia, lymphopenia, and CD4 reduction [39].

Additionally, in the phase II KAMELEON study (NCT02999672), the aim was to explore tumor HER2 expression and its impact on T-DM1 response in patients with HER2-positive urothelial carcinoma (UC), pancreatic cancer, or cholangiocarcinoma. Results showed that some patients with HER2-positive UC or pancreatic cancer could benefit from T-DM1 treatment [40].

### Inotuzumab ozogamicin (InO)

The CD22 antigen, a 135-kDa type I transmembrane sialoglycoprotein, is found in the cytoplasm of nearly all B lineage cells and is specifically expressed on B cells. The CD22 antigen is predominantly expressed in IgM^+^ IgD^+^ B cells [41]. InO is an ADC drug created by conjugating the human IgG4 mAb targeting CD22 with the cytotoxic chemotherapeutic drug calicheamicin through an acid-unstable splice. The binding of InO to CD22-expressing tumor cells initiates endocytosis of the InO-CD22 complex, leading to hydrolysis of the N-acetyl-γ-khakimycin dimethylhydrazide junction. Activation of N-acetyl-γ-kadzimycin dimethylhydrazide induces double-stranded DNA breaks, subsequently causing cell cycle arrest and cell death [42]. InO plays a crucial role in the treatment of acute lymphoblastic leukemia (ALL) by targeting cancer cells that abnormally express CD22, thereby inducing cell cycle arrest and apoptosis [43].

InO seems to be an effective salvage measure for patients with advanced ALL, enabling more patients to undergo stem cell transplantation and achieve long-term survival [44]. A phase III trial of InO in relapsed/refractory ALL has been completed. In this study, patients treated with InO exhibited significantly higher CR rates, lower disease burden in remission, and longer duration of remission compared to the group treated with standard chemotherapy [45]. Meanwhile, patients treated with InO showed improved clinical outcomes and QoL [46]. A multicenter, parallel, open-label phase III trial was conducted to assess the efficacy of InO in adult patients with recurrent/refractory ALL. The results indicated a higher CR or CR with incomplete hematologic recovery rate in the InO group compared to the standard-of-care (SoC) group. The median OS was 7.7 months in the InO group and 6.2 months in the SoC group [47]. In a study evaluating the antitumor activity and safety of InO for the treatment of CD22-positive relapsed/refractory ALL, the results showed that all treated patients had a median PFS of 3.9 months and a median OS of 7.4 months. The most common AEs with any grade included neutropenia (28%), increased AST (26%), nausea (21%), vomiting (17%), fatigue (15%), and febrile neutropenia (15%) [48].

In a phase II trial, InO was investigated as a monotherapy in pediatric patients with relapsed/refractory ALL. The study included a total of 32 enrolled patients, with 28 receiving treatment, and 27 being evaluable for efficacy. The results revealed a 1-year EFS rate of 36.7% and an OS rate of 55.1% [49]. In a multicenter study focusing on low-dose post-transplant InO for preventing relapse in ALL, it was found that the maximum tolerated dose of InO was 0.6 mg/m^2^. The study reported a 1-year non-relapse mortality rate of 5.6%, a PFS of 89%, and an OS of 94% [50].

The detection of measurable residual disease stands out as a significant predictor of relapse in ALL. In a phase II study investigating InO for the palliation of measurable residual disease in ALL, the results indicated a 69% response rate, leading to measurable residual disease negativity. The 2-year relapse-free survival rate for the entire cohort was 54%, and the a 2-year OS rate was 60%. Most AEs were of lowgrade. Consequently, InO demonstrates favorable survival rates, measurable residual disease negativity, and safety for patients with ALL and measurable residual disease positivity [51].

### Moxetumomab pasudotox (MP)

Developed by AstraZeneca and granted FDA approval in 2018, MP is a recombinant immunotoxin comprising moxetumomab targeting CD22, a 38 kDa fragment of pseudomonas exotoxin A, and the linker mc–VC–PABC. It is utilized for treating adult patients with relapsed/refractory hairy cell leukemia (HCL) who have not responded to at least two systemic therapies (including purine nucleoside analogues). MP marks the first drug approved for HCL treatment in over 20 years [52].

The FDA approval of MP relies on data from the phase III clinical study, Study 1053, which was a single-arm, multicenter study involving 80 patients diagnosed with HCL or an HCL variant. These patients had undergone at least two systemic treatments. The treatment involved intravenous injection of 40 µg/kg MP on the 1st, 3rd, and 5th day of each 28-day cycle, totaling 6 cycles. The primary endpoint was CR, defined as achieving CR and maintaining hematologic remission for over 180 days. The data revealed that MP monotherapy achieved an ORR of 75%, a CR of 41%, and a durable CR of 30%. The most common AEs (grade 3–4) included decreased lymphocyte count (20%), asymptomatic hypophosphatemia (10%), and anemia (10%) [53]. Updated data confirmed that MP exhibited high durable response rates and a minimal residual disease negative rate in heavily pre-treated patients with HCL. It was deemed safe, manageable, and a new feasible treatment option [54].

### Polatuzumab vedotin (PV)

Developed by Genentech, PV is an ADC composed of the antibody CD79b linked to MMAE through a cleavable dipeptide linker (mc–VC–PABC). It received its initial approval for the treatment of adult patients with relapsed/refractory diffuse large B-cell lymphoma (DLBCL) who have undergone at least two prior therapies in conjunction with bendamustine and rituximab (BR) [55].

The approval was based on findings from an open-label, global, multicenter, phase Ib/II clinical study known as GO29365. In this study, 80 patients with relapsed/refractory DLBCL, who had previously undergone at least one treatment regimen, were randomly assigned to two groups. One group received BR with PV, while the other group received BR alone. Both groups underwent a total of six 21-day cycles of treatment. The study assessed CR rate as primary endpoint. Results demonstrated a higher CR rate in the BR with PV group compared to the BR alone group, with a significantly elevated CR rate evaluated by the independent review committee at the end of treatment (40.0% vs. 17.5%). In the BR with PV group, the most common grade 3–4 AEs included thrombocytopenia (41%), neutropenia (46.2%), infection and infestation (23.1%), and anemia (28.2%). Additionally, among transplant-ineligible patients with relapsed/refractory DLBCL, the BR with PV group exhibited a 58% lower risk of death compared to the BR group [56]. The phase III POLARIX study (NCT03274492) further demonstrated PV as an effective option for treating patients with relapsed/refractory DLBCL [57]. It revealed that PV in combination with rituximab plus cyclophosphamide, doxorubicin, and prednisone (Pola-R-CHP) significantly improved PFS compared to R-CHOP in both Asian and global populations, with comparable safety profiles between Pola-R-CHP and R-CHOP [58].

A preclinical investigation demonstrated that PV induces the degradation of the BCL-2 protein family member MCL-1 through the ubiquitin/proteasome system. When PV was used in combination with venetoclax and anti-CD20 antibodies obinutuzumab or rituximab, the targeted MCL-1 antagonistic effect led to tumor regression in preclinical non-Hodgkin lymphoma (NHL) models. Importantly, these regressions were sustained even after discontinuation of treatment. In the phase Ib clinical trial, severely pre-treated patients with recurrent or refractory NHL received the combination therapy of PV, venetoclax, and an anti-CD20 antibody. A significant proportion of patients responded positively to the treatment, with 76% of patients with follicular lymphoma and 29% of patients with DLBCL achieving either complete or partial responses [59].

In a phase Ib/II trial evaluating the safety and activity of mosunetuzumab plus PV in relapsed/refractory aggressive large B-cell lymphoma (LBCL), the best ORR was 59.2%, the CR rate was 45.9%, median PFS was 11.4 months, and median OS was 23.3 months. The most common grade ≥ 3 AEs were neutropenia and fatigue. These findings suggest that the combination of mosunetuzumab with PV exhibits good safety and a highly persistent response, making it suitable as a second-line treatment for patients with relapsed/refractory LBCL who are not eligible for transplant [60].

However, a single-arm, phase Ib/II study revealed that PV combined with rituximab and lenalidomide in treating patients with relapsed/refractory DLBCL did not meet the threshold of predetermined activity. The CR rate was 31%, and the most common grade 3–4 AEs were neutropenia and thrombocytopenia [61].

### Enfortumab vedotin (EV)

Nectin-4, a type I transmembrane protein, is notably overexpressed in various malignant tumors, including bladder cancer. Its overexpression plays a role in promoting tumor cell proliferation, differentiation, and invasion through the activation of the PI3K/AKT signaling pathway, contributing to malignant tumorigenesis, metastasis, and recurrence [62, 63]. Consequently, Nectin-4 has emerged as a promising target for systemic therapy in locally advanced or metastatic urothelial carcinoma (la/mUC). EV is an ADC that combines a human antibody against Nectin-4 with the cytotoxic MMAE through a cleavable junction. Upon binding to Nectin-4, EV forms a complex that internalizes within Nectin-4-expressing cells. The released MMAE binds to tubules, disrupting the cellular microtubule network and leading to cell cycle arrest and apoptosis [64].

In the phase I dose-escalation study EV-101, incremental administration of 1.25 mg/kg EV occurred on the 1st, 8th, and 15th day of a 28-day cycle. Results from the study involving 112 patients with mUC treated with single-agent EV showed an investigator-assessed confirmed ORR of 43%, with a DOR lasting 7.4 months. The median OS was 12.3 months, and the 1-year OS rate reached 51.8%. The most frequently reported treatment-related adverse events (TRAEs) with an incidence rate of ≥ 30% included fatigue, alopecia, decreased appetite, dysgeusia, nausea, peripheral sensory neuropathy, pruritus, and diarrhea [65].

In a two-cohort, single-arm, phase II study (EV-201) involving 125 patients with metastatic UC, a final ORR of 44%, a CR rate of 12%, and a median DOR of 7.6 months were confirmed. This demonstrated a more favorable treatment outcome compared to standard chemotherapy [66]. The EV-301 trial further showcased the ability of EV to prolong the OS of patients compared to standard chemotherapy, with a 30% reduction in the risk of death, as indicated by a hazard ratio (HR) of 0.70 (95% confidence interval[CI] 0.58–0.85). PFS also improved with EV compared to chemotherapy, with an HR of 0.63 (95% CI 0.53–0.76). The incidence of TRAEs was 93.9% for EV and 91.8% for chemotherapy, with the incidence rates of grade ≥ 3 AEs being 52.4% and 50.5%, respectively. AEs associated with EV were manageable [67].

Subsequent retrospective studies of EV monotherapy have demonstrated its effectiveness in treating individuals in important patient populations previously excluded from clinical trials, including those with conditions such as diabetes. This highlights the maturation of research on EV with a broader population of recipients [68].

The FDA granted breakthrough therapy designation to the combination of EV with pembrolizumab (EV + P), approving it as a first-line treatment for patients with la/mUC who are not suitable for cisplatin [64]. In a phase II trial study, cisplatin-ineligible patients received treatment with EV + P, leading to demonstrated tumor shrinkage in a majority of the patients [69]. EV + P showcased the preservation or improvement of QoL, functioning, and symptoms in cisplatin-ineligible patients with la/mUC. Notable and clinically meaningful improvements were observed in European Organization for Research and Treatment of Cancer Quality of Life Questionnaire-Core Questionnaire (EORTC QLQ-C30) scores at weeks 12 and weeks 24 in the EV + P group. Additionally, there was a significant decrease in worst pain scores measured by the Brief Pain Inventory Short Form (BPI-SF). In patients receiving EV monotherapy, the overall QoL assessed by the EORTC QLQ-C30 remained stable. These findings suggest that both EV + P and EV monotherapy have a positive impact on QoL, functioning, and symptom management in patients with la/mUC who are ineligible for cisplatin-based therapy [70].

In another study involving patients with la/mUC ineligible for cisplatin therapy, EV + P demonstrated a high confirmed ORR and a persistent response as first-line therapy [71].

### Trastuzumab deruxtecan (T-DXd)

T-DXd is an ADC that combines trastuzumab (a humanized mAb targeting HER2) with an exatecan derivative (a topoisomerase I inhibitor) through a linker designed for the targeted delivery of cytotoxic agents into cancer cells. In comparison to T-DM1, T-DXd can deliver a higher payload of cytotoxic drugs, and its improved membrane permeability allows it to kill more tumor cells through the “bystander effect” [72, 73]. The FDA has approved T-DXd for the treatment of adult patients with unresectable or metastatic HER2-positive breast cancer who have previously received two or more anti-HER2 therapies [74].

In an open-label, dose-escalation, and dose-expansion phase I trial, investigators assessed the safety, tolerability, and activity of T-DXd in advanced solid tumors expressing HER2. The results revealed a confirmed objective response in 66 out of 111 patients, with disease control confirmed in 104 out of 111 patients. The median follow-up was 9.9 months, and the median time to response, DOR, and PFS were 1.6 months, 20.7 months, and 22.1 months, respectively. All patients experienced at least one TEAE. Common grade 3 or more severe TEAEs included anemia (17%), neutropenia (14%), leukopenia (9%), and thrombocytopenia (8%). Moreover, 19% of patients experienced at least one serious TEAE, and interstitial lung disease, organizing pneumonia, or pneumonitis occurred in 20 patients [75].

In the phase II DESTINY-Breast01 trial, T-DXd demonstrated sustained antitumor activity in patients with HER2-positive metastatic breast cancer who had previously received ≥ 2 anti-HER2 treatments, including T-DM1 [76]. Subgroup analysis from DESTINY-Breast01 indicated markedly improved patient outcomes when T-DXd was used to treat HER2-positive metastatic breast cancer, showcasing durable efficacy even in cases with brain metastases [77]. Updated results further supported the evidence that T-DXd maintains sustained antitumor activity and consistent safety in HER2-positive metastatic breast cancer with brain metastases [78]. In the open-label, single-arm phase II trial TUXEDO-1, designed for patients with HER2-positive breast cancer with brain metastases after prior therapy, T-DXd demonstrated a high response rate in these patients [79].

In DESTINY-Breast02, a randomized phase III trial, patients with HER2-positive metastatic breast cancer who had progressed after a trastuzumab-containing regimen were randomly assigned to two groups: one receiving T-DXd treatment, and the other receiving treatment of the physician’s choice. The median PFS was 17.8 months in the T-DXd group compared to 6.9 months in the treatment of the physician’s choice group. The most common TEAEs were nausea in both groups (73% vs. 37%). However, more grade 3 or worse TEAEs occurred in the T-DXd group (53% vs. 44%) [80]. In the DESTINY-Breast03 phase III trial, T-DXd demonstrated a significant improvement in OS compared to T-DM1 in HER2-positive metastatic breast cancer patients. T-DXd also displayed a manageable safety profile and a longer treatment duration [81, 82]. Additionally, based on the outcomes of the DESTINY-Breast04 trial [83], T-DXd was approved as the first therapy for the treatment of HER2-low metastatic breast cancer [84].

### Sacituzumab govitecan (SG)

Trophoblast cell surface antigen 2 (Trop-2), a 40-kDa glycoprotein also known as tumor-associated calcium signal transducer-2, plays a crucial role in the development and metastasis of various solid tumors [85]. SG comprises an anti-Trop-2 antibody, a SN-38 payload (an active metabolite of irinotecan), and a CL2A linker. Noteworthy features of SG include the use of the moderately toxic drug SN-38, the utilization of a moderately stable conjugate, and a high drug‒antibody ratio (7–8:1), resulting in low off-target toxicity [86]. In April 2020, SG received accelerated approval from the FDA for the treatment of adult patients with metastatic triple-negative breast cancer (TNBC) who have undergone at least two prior treatments for metastatic disease [87].

In the initial clinical trial of SG, it demonstrated encouraging antitumor activity in patients with metastatic solid tumors, including pancreatic cancer, TNBC, colorectal cancer, small cell lung cancer (SCLC), gastric cancer (GC), UC, among others [88]. In a single-arm, multicenter trial, SG was administered to 108 patients with metastatic TNBC who had undergone at least two anticancer treatments before. Results revealed a median DOR of 7.7 months, PFS of 5.5 months, and OS of 13.0 months. The most common AEs included nausea, neutropenia, diarrhea, fatigue, and anemia [89]. The TROPiCS-02 study, a randomized, multicenter, phase III trial, evaluated the efficacy of SG in patients with pretreated, endocrine-resistant hormone receptor-positive, HER2-negative metastatic breast cancer. Among the 543 patients randomized into the SG group (*n* = 272) and the chemotherapy group (*n* = 271), the median OS was significantly longer in the SG group (14.4 months) compared to the chemotherapy group (11.2 months). The ORR in the two groups was 21% and 14%, respectively [90]. The NeoSTAR trial assessed the efficacy and feasibility of neoadjuvant SG in patients with localized TNBC. In this trial, 98% of patients completed four rounds of SG, 30% achieved partial CR, and 64% achieved ORR. Common AEs included nausea, fatigue, alopecia, neutropenia, and rash [91].

SG received FDA approval as a second-line treatment for patients with la/mUC who had previously received platinum and PD-1/PD-L1 inhibitors [86]. In the IMMU-132-01 trial, among the 45 patients with mUC, the ORR was 31%. Specifically, in patients with visceral involvement, the ORR was 27%, and in those who had previously received immune checkpoint inhibitors (ICIs) treatment, the ORR was 23%. The clinical benefit rate for all patients was 47%, with a median DOR of 12.9 months, median PFS of 7.3 months, and median OS of 16.3 months. These findings indicate that SG demonstrates clinical activity in patients with relapsed/refractory mUC, including those previously treated with ICIs and those with visceral disease [92].

The TROPHY-U-01 trial, an open, multi-cohort phase II clinical trial (NCT03547973), aimed at evaluating the efficacy and safety of SG treatment in patients with locally advanced or metastatic UC who had failed platinum- and ICI-based therapies. Among the 113 patients receiving SG (10 mg/kg body weight) on the 1st and 8th day of a 21-day cycle, the ORR was 27%, with median DOR, PFS, and OS of 7.2 months, 5.4 months, and 10.9 months, respectively. The main grade ≥ 3 TRAEs included neutropenia, leukopenia, anemia, diarrhea, and febrile neutropenia [93]. Updated data confirmed a sustained high ORR with longer follow-up (28%), and median PFS, OS, and TRAEs were consistent with previous outcomes [94]. Moreover, some studies suggest that cells resistant to EV remain sensitive to SG, making SG potentially effective in most subtypes of bladder cancer, including those treated with EV, marking a significant development in the treatment of patients with UC [95]. In a phase I trial evaluating the safety and efficacy of SG plus EV in mUC, results showed an impressive ORR of 70%, with grade ≥ 3 AEs occurring in 78% of patients. The most common grade ≥ 3 TRAEs included neutropenia, anemia, urinary tract infection, fatigue, and diarrhea [96].

### Disitamab vedotin (RC48)

RC48, developed by RemeGen, represents the third marketed HER2-targeted ADC. It comprises a novel humanized HER2 antibody, a histone-cleavable linker (mc–VC–PABC), and a cytotoxic agent (i.e., MMAE) [97]. In June 2021, the National Medical Products Administration granted approval for RC48 as a treatment for patients with locally progressive or metastatic GC (including gastric junction adenocarcinoma) who exhibit HER2 overexpression and have undergone at least two rounds of systemic chemotherapy [98]. Notably, it is the inaugural ADC drug approved for marketing in China.

In a dose-escalating, dose-expanding phase I clinical trial (NCT02881190), RC48 demonstrated promising safety and antitumor activity in HER2-positive solid tumors. The study results revealed dose-dependent antitumor activity, showcasing an ORR of 21.0%, with a PFS of 3.5 months. Common grade 3 and higher AEs included neutropenia, leukopenia, hyperalgesia, and elevated bound blood bilirubin [99].

In a single-arm Phase II clinical trial (NCT03556345), which enrolled 125 patients with HER2-positive locally advanced or metastatic GC (including gastroesophageal junction adenocarcinoma) previously treated with second-line or higher regimens, the study reported an ORR of 24.8%, a PFS of 4.1 months, a median time to disease progression of 4.2 months, and an OS of 7.9 months. The most frequent AEs included decreased white blood cell count, asthenia, hair loss, decreased neutrophil count, and others [100].

An observational multicenter real-world study enrolled 45 cases of advanced and metastatic GC with a history of failure with two or more prior therapies. Patients were subjected to either RC48 monotherapy or a combination of RC48 and ICIs as third-line therapy until disease progression, death, or intolerable toxicity ensued. Both groups received an intravenous injection at a dose of 2.5 mg/kg every 2 weeks. In the RC48 plus ICIs treatment group, tislelizumab was intravenously administered at a dose of 200 mg every 3 weeks. The results revealed an ORR and disease control rate (DCR) of 24.4% (11/45) and 66.7% (30/45), respectively. Patients treated with RC48 in combination with ICIs demonstrated a superior ORR (36.0% vs. 10.0%) and DCR (80.0% vs. 50.0%) compared to those receiving RC48 monotherapy. Additionally, the median PFS in the RC48 plus ICIs treatment group surpassed that in the RC48 monotherapy group (6.2 months vs. 3.9 months). This study illustrated that the combination of ICIs with RC48 exhibited superior therapeutic efficacy as a third-line or later treatment in patients with HER2-positive or HER2-low advanced and metastatic GC. Importantly, this combined treatment demonstrated a manageable safety profile compared to RC48 monotherapy [101].

In recent years, ADC agents have showcased compelling efficacy and survival advantages for patients with la/mUC [102, 103]. In a phase II clinical study (RC48-C005), 43 patients with HER2-positive la/mUC that had progressed after at least one prior systemic chemotherapy were enrolled. The study results demonstrated an ORR of 51.2%, a DCR of 90.7%, a median PFS of 6.9 months, and an OS of 13.9 months. The most frequently observed TRAEs included hypoesthesia, alopecia, and leukopenia [104]. Based on these findings, RC48 has received approval as a second-line treatment for patients with mUC who have experienced progression after receiving platinum-based chemotherapy and exhibit HER2 overexpression. A combined analysis of two phase II clinical trials (RC48-C005 and RC48-C009) assessed the safety and efficacy of RC48 in patients with HER2-positive la/mUC refractory to at least one prior systemic chemotherapy. The confirmed ORR was 50.5%, and the median DOR, PFS, and OS were 7.3 months, 5.9 months, and 14.2 months, respectively. The most common TRAEs included peripheral sensory neuropathy, leukopenia, increased glutamic oxaloacetic transaminase, and neutropenia [105].

To date, multiple studies have assessed the efficacy and safety of combining RC48 with immunotherapy for la/mUC). In a retrospective, multicenter study involving 36 patients with la/mUC, the median PFS in the RC48 alone group was 5.4 months, while in the RC48 plus immunotherapy group, it was 8.5 months. The primary TRAEs included anemia, hypoesthesia, fatigue, and elevated transaminase [106].

In a two-center real-world study, nine patients with la/mUC received intravenous injections of RC48 along with tislelizumab or toripalimab, resulting in a confirmed ORR of 88.9%. CRs were observed in five patients, and the median radiological PFS was 12.0 months [107]. Another retrospective, multicenter study reported an ORR of 63.2% and a DCR of 89.5% in patients with la/mUC who received tislelizumab in combination with RC48. The median PFS was 8.2 months, and the median DOR was 7.3 months. Common TRAEs included anemia, anorexia, asthenia, hypoesthesia, and others [108].

The HOPE-03 study, a multicenter, single-arm, phase Ib/II trial, aimed to evaluate the safety and efficacy of combining RC48 with tislelizumab as novel neoadjuvants in patients with HER2-positive la/mUC. The dose-escalation phase of the study recommended a dosage of 2.0 mg/kg for RC48 in the phase II stage, and a total of 45 patients were included in the phase II study [109].

### Loncastuximab tesirine (LT)

LT is an ADC with a CD19-targeting mechanism. It comprises a humanized anti-human CD19 mAb connected to a pyrrolobenzodiazepine dimer toxin through a valine-alanine linker. This ADC was approved for treating patients with relapsed/refractory LBCL who have undergone second-line or above systemic treatment, encompassing unspecified DLBCL, DLBCL caused by low-grade lymphoma, and high-grade B-cell lymphoma [110].

An open, single-arm, phase I study aimed to investigate the safety and tolerability of LT. The results indicated that LT exhibited high single-agent antitumor activity and an acceptable safety profile in patients with NHL [111]. In a subsequent single-arm, phase II clinical trial evaluating LT monotherapy in patients with relapsed/refractory DLBCL (LOTIS-2, NCT03589469), the ORR was 48.3%, with a median DOR of 10.3 months, a median PFS of 4.9 months, and a median OS of 9.9 months. The most common TEAEs included neutropenia, thrombocytopenia, and increased gamma-glutamyltransferase, and 39% of patients experienced serious AEs, including neutropenia, pleural effusion, anemia, pericardial effusion, and non-cardiac chest pain [112]. Updated analyses from LOTIS-2 revealed that 48.3% of patients achieved an ORR, with 24.8% achieving a CR, and the median OS for all treated patients was 9.5 months. Grade ≥ 3 TEAEs occurred in 73.8% of the patients [113]. Moreover, similar responses were observed in both younger (age < 70 years old) and older (age ≥ 70 years old) groups. The ORR was 48.4% in the younger group and 48.0% in the older group. The median time to CR for the young and older groups was 42 days and 41 days, respectively [114].

### Tisotumab vedotin (TV)

Tissue factor (TF), also known as thrombospondin kinase, coagulation factor III, or CD142, is a transmembrane glycoprotein whose primary function is to initiate the exogenous coagulation pathway [115]. TF is often expressed on the surface of cancer cells and plays an essential role in tumor growth, angiogenesis and metastasis. TF is aberrantly expressed in a variety of solid tumors, including cervical cancer, breast cancer, ovarian cancer, non-small cell lung cancer (NSCLC), and glioblastoma [116]. TV contains a fully human mAb targeting TF that is conjugated with MMAE. TV was approved for the treatment of recurrent or metastatic cervical cancer (r/mCC) in adult patients with a disease progression during or after chemotherapy [117].

The safety, tolerability, pharmacokinetic profile and antitumor activity of TV were evaluated in LA and/or metastatic solid tumors with TF expresssion in a phase I/II open-label, dose-escalation, and extension study (innovaTV201; NCT02001623). Patients were those with recurrent, advanced or metastatic ovarian cancer, endometrial cancer, cervical cancer, prostate cancer, bladder cancer, oesophageal cancer, head and neck squamous cell cancer or NSCLC. The dose-escalation phase showed a maximum tolerated dose of 2.0 mg/kg. The dose-expansion phase showed an ORR of 15.6% for all tumor types, with an ORR of 24% in the cervical cancer group. The median DOR and PFS was 5.7 months and 3.0 months, respectively. Any grade of TEAEs included epistaxis, fatigue, nausea, alopecia, conjunctivitis, et al [118]. InnovaTV 204 (NCT03438396) was a multicenter, single-arm, phase II study in which a total of 101 patients with r/mCC received at least one intravenous injection of TV. The results showed an ORR of 24%, a DCR of 72%, and a median OS of 12.1 months. The most common TRAEs were alopecia, epistaxis, nausea, conjunctivitis, fatigue and dry eye [119]. Based on this study, TV was approved for second-line treatment of r/mCC.

The innovaTV 206 study, a single-arm, open-label phase I/II trial, assessed the safety and efficacy of TV in Japanese patients with recurrent or metastatic cervical cancer (r/mCC). The confirmed ORR was 29.4%, with a median DOR of 7.1 months and a median time to response of 1.2 months. The most frequently reported TEAEs included anemia, nausea, alopecia, epistaxis, and diarrhea [120]. The innovaTV 205/GOG-3024/ENGOT-cx8 Study, an open-label, multicenter phase Ib/II clinical trial (NCT03786081), included 41 r/mCC patients in the dose-escalation study and 101 patients in the dose-expansion study. Patients received TV in combination with bevacizumab, pembrolizumab, or carboplatin. Results indicated an ORR of 54.5% with first-line TV + carboplatin, 40.6% with first-line TV + pembrolizumab, and 35.3% with second-line/third-line TV + pembrolizumab. The median DOR was 8.6 months and 14.1 months in the first-line TV + carboplatin group and the second-line/third-line TV + pembrolizumab group, respectively [121].

### Mirvetuximab soravtansin (MIRV)

Folate receptor α (FRα) possesses a high affinity for folate, facilitating its transport to the cytoplasm through endocytosis. While FRα is typically expressed at low levels in normal tissues [122, 123], it is often abnormally expressed in various epithelial tumors, including epithelial ovarian cancer, endometrial adenocarcinoma, TNBC, and NSCLC [124–126]. MIRV, an ADC, employs a FRα-targeted antibody linked to a microtubule inhibitor via a cleavable linker. It received approval for treating adult patients with positive FRα expression, platinum-resistant epithelial ovarian, fallopian tube, or primary peritoneal cancer who have previously undergone 1–3 systemic treatment regimens [127, 128].

In a phase I expansion clinical trial (NCT01609556), 46 patients with platinum-resistant epithelial ovarian, fallopian tube, or primary peritoneal cancer and positive FRα expression were administered MIRV once every 3 weeks at a dose of 6.0 mg/kg. Results revealed a confirmed ORR of 26%, a median PFS of 4.8 months, and a median DOR of 19.1 weeks. Common TRAEs included diarrhea, blurred vision, nausea, and fatigue [129]. Another phase I expansion study demonstrated an ORR of 31% and a PFS of 5.4 months in recurrent ovarian cancer with the highest FRα expression level [130].

The SORAYA study, a single-arm, phase II study, assessed the safety and efficacy of MIRV in patients with platinum-resistant epithelial ovarian cancer and high FRα expression who had undergone 1–3 prior therapies. Results showed an ORR of 32.4% and a median DOR of 6.9 months. The most common TEAEs of MIRV were blurred vision, keratoconus, and nausea [131].

In a randomized, phase III study involving 366 patients randomized into the MIRV group (*n* = 243) and the chemotherapy group (*n* = 109), MIRV did not significantly improve PFS compared to chemotherapy. However, fewer TRAEs were observed in the MIRV group than in the chemotherapy group [132]. In a subsequent global, open-label, controlled trial, the MIRV group exhibited a median PFS of 5.62 months compared to 3.98 months in the chemotherapy group. The ORR in the MIRV group was significantly higher (42.3% vs. 15.9%), and the median OS in the MIRV group was 16.46 months, while in the chemotherapy group, it was 12.75 months [133].

Currently, ongoing studies are exploring the safety and efficacy of combining MIRV with other drugs in patients with ovarian cancer. In a phase Ib escalation study, the safety and antitumor activity of MIRV plus carboplatin in the treatment of relapsed, platinum-sensitive epithelial ovarian or fallopian tube cancer patients were evaluated. The study demonstrated a confirmed ORR of 71%, with a median PFS of 15 months [134].

Another phase Ib study assessed the safety and efficacy of the combination therapy of MIRV and bevacizumab in platinum-resistant ovarian cancer patients with positive FRα expression. The study revealed a confirmed ORR of 39% and a median PFS of 6.9 months. The most common TRAEs included diarrhea, blurred vision, nausea, and fatigue [135]. Additionally, in a cohort of 94 patients with platinum-resistant ovarian cancer treated with MIRV and bevacizumab, the ORR was 44%, with a median PFS of 8.2 months and a median DOR of 9.7 months. The most frequently observed TRAEs were blurred vision, diarrhea, and nausea [136].

**Table 1:** Overview of FDA-approved ADC drugs

company	generation	ADC drug (brand name)	target	isotype	antibody	linker	payload	average DAR	tumor types	approval date
Pfizer	First	Gemtuzumab Ozogamicin (Mylotarg)	CD33	IgG4κ	Gemtuzumab	hydrazone	Calicheamicin	2–3	AML	May 17, 2000 Sep 1, 2017
Seagen, Takeda	Second	Brentuximab Vedotin (Adcetris)	CD30	IgG1κ	Brentuximab	mc‒VC‒PABC	MMAE	4	HL, ALCL	Aug 19, 2011
Genentech	Second	Trastuzumab Emtansine (Kadcyla)	HER2	IgG1κ	Trastuzumab	SMCC	DM1	3.5	Breast cancer	Feb 22, 2013
Pfizer	Second	Inotuzumab Ozogamicin (Besponsa)	CD22	IgG4κ	Inotuzumab	hydrazone	Calicheamicin	5–7	ALL	Jun 28, 2017 (EMA) Aug 17, 2017 (FDA)
AstraZeneca	Second	Moxetumomab Pasudotox (Lumoxiti)	CD22	IgG4κ	Moxetumomab	mc‒VC‒PABC	PE38	1.8	HCL	Sep 13, 2018
Genentech	Third	Polatuzumab Vedotin (Polivy)	CD79b	IgG1κ	Polatuzumab	mc‒VC‒PABC	MMAE	3.5	DLBCL	Jun 10, 2019
Astellas, Seagen	Third	Enfortumab Vedotin (Padcev)	Nectin-4	IgG1κ	Enfortumab	mc‒VC‒PABC	MMAE	3.8	UC	Dec 18, 2019
AstraZeneca, Daiichi Sankyo	Third	Trastuzumab Deruxtecan (Enhertu)	HER2	IgG1κ	Trastuzumab	tetrapeptide	DXD	7–8	Breast cancer	Dec 20, 2019
Gilead Sciences	Third	Sacituzumab Govitecan (Trodelvy)	TROP2	IgG1κ	Sacituzumab	CL2A	SN-38	7.6	Breast cancer UC	Apr 22, 2020
RemeGen	Third	Disitamab Vedotin (Aidixi)	HER2	IgG1κ	Disitamab	mc‒VC‒PABC	MMAE	4	GC UC	Jun 8, 2021 (NMPA)
ADC Therapeutics	Third	Loncastuximab Tesirine (Zynlonta)	CD19	IgG1κ	Loncastuximab	mc‒VC‒PABC	PBD SG3199	2.3	DLBCL	Apr 23, 2021
Seagen	Third	Tisotumab Vedotin (Tivdak)	TF	IgG1κ	Tisotumab	mc‒VC‒PABC	MMAE	4	Cervical cancer	Sep 20, 2021
ImmunoGen	Third	Mirvetuximab Soravtansine (Elahere)	FRα	IgG1κ	Mirvetuximab	Sulfo-SPDB	DM4	3.3-5	Ovarian cancer	Nov 14, 2022

## Conclusion and future perspectives

ADCs have undergone three generations of technological changes. The first-generation ADCs, represented by Mylotarg, contain murine or chimeric antibodies with unstable linkers, low titer strength of coupled cytotoxic drugs and random coupling, so the effectiveness is not high, and toxic side effects are substantial. Second-generation ADCs, such as BV and T-DM1, contain humanized mAbs that are more stable in their linkers but still cause off-target toxicity in random-coupled connections. Third-generation ADCs use fully humanized antibodies coupled with more efficient cytotoxic drugs and site-conjugation technology to achieve better efficacy, but the toxic side effects of highly toxic drug delivery still exist [137].

Off-target effects can occur due to a single drug, either standardized chemotherapy or mAb therapy. The previous ADC coupling methods and the selection of cytotoxic drugs have been improved, and the selection of composition and mode of component binding is gradually maturing with further exploration. Optimizing dosage and reducing drug side effects and resistance, which are also the ultimate challenges that must be overcome in the clinical use of these drugs, are critical to ensuring the safety and widespread use of ADCs. In addition to their use as a single drug, ADCs in combination with monoclonal antibodies, ICIs or chemotherapy drugs have also attracted much attention [22, 58, 138].

Despite decades of development, treatment with ADCs still has much room for improvement. When ADCs enter the body, the rate at which antibodies penetrate into tissues from plasma is slower relative to the rate of small molecules, and the number of antigens on the surface of the target cells limits the number of antibodies retained in tumor tissue [139]. In fact, multiple studies have shown that the percentage of effector molecules delivered by ADCs to target cells is much less than 1%, with the most optimistic estimate being as low as 1.5% [140]. The average drug-to-antibody ratio (DAR) of most current clinical ADCs is limited to 3.5-4, so the amount of drug delivered by ADCs to tumor cells is low. Many of the cytotoxic drugs used in ADCs are hydrophobic and tend to induce antibody aggregation, which should be avoided to ensure a long shelf life and limit the use of the drug [141]. Increasing the hydrophilicity of cytotoxic metabolites, for example, through charged groups, can reduce the rate of transmembrane transfer, thereby increasing cell retention while minimizing the bystander effect [142, 143]. In addition, cytotoxic drugs are also a difficult problem in ADC research, and conventional chemotherapy drugs are not powerful enough to act as ADC payloads [141].

In terms of ADC coupling methods, the non-site-specific coupling method is the method used in early ADC research; it offers poor stability, easy aggregation, and nontherapeutic toxic side effects due to the easy shedding of cytotoxins and a narrow therapeutic window. Fixed-point coupling technology usually requires modification of antibodies to improve the uniformity of ADCs, and the ADCs obtained by this technology have a suitable DAR, which increases the therapeutic window. Fixed-point coupling will become the trend of ADC development and innovation in the future [144, 145]. As an important direction of therapeutic agent, ADC has received increasing attention and is becoming a key means of cancer treatment. With ongoing improvements related to targeting, reducing toxicity from off-target effects, reducing drug resistance and stability of joints, we believe that ADCs are likely to play an enormous role in tumor therapy in the future.

## Data Availability

No datasets were generated or analysed during the current study.

## Abbreviations

ADCs: antibody‒drug conjugates.
FDA: Food and Drug Administration.
AML: acute myeloid leukemia.
mAb: monoclonal antibodies.
GO: gemtuzumab ozogamicin.
SWOG: Southwest Oncology Group.
ALFA: acute leukemia French association.
AEs: adverse events.
OS: overall survival.
EFS: event-free survival.
BSC: best supportive care.
TEAEs: treatment-emergent adverse events.
BV: brentuximab vedotin.
MMAE: monomethyl auristatin E.
HL: Hodgkin lymphoma.
ALCL: anaplastic large cell lymphoma.
DOR: duration of response.
MF: mycosis fungoides.
C-ALCL: cutaneous anaplastic large cell lymphoma.
PTCL: peripheral T-cell lymphomas.
ORR: objective response rate.
PFS: progression-free survival.
CR: complete response.
T-DM1: trastuzumab emtansine.
DM1: emtansine.
UC: urothelial carcinoma.
InO: inotuzumab ozogamicin.
ALL: acute lymphoblastic leukemia.
SoC: standard-of-care.
MP: moxetumomab pasudotox.
HCL: hairy cell leukemia.
PV: polatuzumab vedotin.
DLBCL: diffuse large B-cell lymphoma.
BR: bendamustine and rituximab.
NHL: non-hodgkin lymphoma.
LBCL: large B cell lymphoma.
EV: enfortumab vedotin.
la/mUC: locally advanced or metastatic urothelial.
QOL: quality of life.
BPI-SF: brief Pain Inventory Short Form.
T-DXd: trastuzumab deruxtecan.
SG: sacituzumab govitecan.
Trop-2: trophoblast cell surface antigen 2.
TNBC: triple-negative breast cancer.
CRC: colorectal cancer.
SCLC: small cell lung cancer.
GC: gastric cancer.
ICIs: immnune checkpoint inhibitors.
TRAEs: treatment-related adverse events.
RC48: disitamab vedotin.
NMPA: National Medical Products Administration.
DCR: disease control rate.
TV: tisotumab vedotin.
TF: tissue factor.
NSCLC: non-small cell lung cancer.
r/mCC: recurrent or metastatic cervical cancer.
LT: loncastuximab tesirine.
PBD: pyrrolobenzodiazepine.
MIRV: mirvetuximab soravtansin.
FRα: folate receptor α.
PE38: pseudomonas exotoxin A.
DM4: maytansinoid.
DXD: deruxtecan.
DAR: drug-to-antibody ratio.

## References

[1] Sung H, Ferlay J, Siegel RL, Laversanne M, Soerjomataram I, Jemal A, Bray F (2021). Global Cancer statistics 2020: GLOBOCAN estimates of incidence and Mortality Worldwide for 36 cancers in 185 countries. CA Cancer J Clin.

[2] Zeng Y (2018). Advances in mechanism and treatment strategy of cancer. Cell Mol Biol (Noisy-le-grand).

[3] Li WQ, Guo HF, Li LY, Zhang YF, Cui JW (2021). The promising role of antibody drug conjugate in cancer therapy: combining targeting ability with cytotoxicity effectively. Cancer Med.

[4] Norsworthy KJ, Ko CW, Lee JE, Liu J, John CS, Przepiorka D, Farrell AT, Pazdur R (2018). FDA approval Summary: Mylotarg for treatment of patients with relapsed or refractory CD33-Positive Acute Myeloid Leukemia. Oncologist.

[5] Fu Z, Li S, Han S, Shi C, Zhang Y (2022). Antibody drug conjugate: the biological missile for targeted cancer therapy. Signal Transduct Target Ther.

[6] Drago JZ, Modi S, Chandarlapaty S (2021). Unlocking the potential of antibody-drug conjugates for cancer therapy. Nat Rev Clin Oncol.

[7] Tsuchikama K, An Z (2018). Antibody-drug conjugates: recent advances in conjugation and linker chemistries. Protein Cell.

[8] Gottardi M, Sperotto A, Ghelli Luserna Di Rorà A, Padella A, Cangini D, Giannini MB, Simonetti G, Martinelli G, Cerchione C (2020). Gemtuzumab ozogamicin in acute myeloid leukemia: past, present and future. Minerva Med.

[9] van Der Velden VH, te Marvelde JG, Hoogeveen PG, Bernstein ID, Houtsmuller AB, Berger MS, van Dongen JJ (2001). Targeting of the CD33-calicheamicin immunoconjugate mylotarg (CMA-676) in acute myeloid leukemia: in vivo and in vitro saturation and internalization by leukemic and normal myeloid cells. Blood.

[10] Larson RA, Sievers EL, Stadtmauer EA, Löwenberg B, Estey EH, Dombret H, Theobald M, Voliotis D, Bennett JM, Richie M (2005). Final report of the efficacy and safety of gemtuzumab ozogamicin (mylotarg) in patients with CD33-positive acute myeloid leukemia in first recurrence. Cancer.

[11] Petersdorf SH, Kopecky KJ, Slovak M, Willman C, Nevill T, Brandwein J, Larson RA, Erba HP, Stiff PJ, Stuart RK (2013). A phase 3 study of gemtuzumab ozogamicin during induction and postconsolidation therapy in younger patients with acute myeloid leukemia. Blood.

[12] Castaigne S, Pautas C, Terré C, Raffoux E, Bordessoule D, Bastie JN, Legrand O, Thomas X, Turlure P, Reman O (2012). Effect of gemtuzumab ozogamicin on survival of adult patients with de-novo acute myeloid leukaemia (ALFA-0701): a randomised, open-label, phase 3 study. Lancet.

[13] Amadori S, Suciu S, Selleslag D, Aversa F, Gaidano G, Musso M, Annino L, Venditti A, Voso MT, Mazzone C (2016). Gemtuzumab Ozogamicin Versus Best supportive care in older patients with newly diagnosed Acute myeloid leukemia unsuitable for intensive chemotherapy: results of the Randomized Phase III EORTC-GIMEMA AML-19 Trial. J Clin Oncol.

[14] Appelbaum FR, Bernstein ID (2017). Gemtuzumab ozogamicin for acute myeloid leukemia. Blood.

[15] Lamba JK, Chauhan L, Shin M, Loken MR, Pollard JA, Wang YC, Ries RE, Aplenc R, Hirsch BA, Raimondi SC (2017). CD33 splicing polymorphism determines Gemtuzumab Ozogamicin response in De Novo Acute myeloid leukemia: Report from Randomized Phase III Children’s Oncology Group Trial AAML0531. J Clin Oncol.

[16] Lambert J, Pautas C, Terre C, Raffoux E, Turlure P, Caillot D, Legrand O, Thomas X, Gardin C, Gogat-Marchant K (2019). Gemtuzumab ozogamicin for de novo acute myeloid leukemia: final efficacy and safety updates from the open-label, phase III ALFA-0701 trial. Haematologica.

[17] Dohner H, Weber D, Krzykalla J, Fiedler W, Kuhn MWM, Schroeder T, Mayer K, Lubbert M, Wattad M, Gotze K (2023). Intensive chemotherapy with or without gemtuzumab ozogamicin in patients with NPM1-mutated acute myeloid leukaemia (AMLSG 09–09): a randomised, open-label, multicentre, phase 3 trial. Lancet Haematol.

[18] Pawinska-Wasikowska K, Czogala M, Skoczen S, Surman M, Rygielska M, Ksiazek T, Pac A, Wieczorek A, Skalska-Sadowska J, Samborska M (2023). Gemtuzumab ozogamicin for relapsed or primary refractory acute myeloid leukemia in children-the Polish Pediatric Leukemia and Lymphoma Study Group experience. Front Immunol.

[19] Freeman SD, Thomas A, Thomas I, Hills RK, Vyas P, Gilkes A, Metzner M, Jakobsen NA, Kennedy A, Moore R (2023). Fractionated vs single-dose gemtuzumab ozogamicin with determinants of benefit in older patients with AML: the UK NCRI AML18 trial. Blood.

[20] Montesinos P, Kota V, Brandwein J, Bousset P, Benner RJ, Vandendries E, Chen Y, McMullin MF (2023). A phase IV study evaluating QT interval, pharmacokinetics, and safety following fractionated dosing of gemtuzumab ozogamicin in patients with relapsed/refractory CD33-positive acute myeloid leukemia. Cancer Chemother Pharmacol.

[21] Awada H, Abdelmalek M, Cronin T, Baron J, Kashour Z, Azad F, Faisal MS, Faber M, Gravina M, Sung PJ (2023). Gemtuzumab ozogamicin plus standard induction hemotherapy improves outcomes of newly diagnosed intermediate cytogenetic risk acute myeloid leukemia. Blood Cancer J.

[22] Russell NH, Wilhelm-Benartzi C, Othman J, Dillon R, Knapper S, Batten LM, Canham J, Hinson EL, Betteridge S, Overgaard UM et al. Fludarabine, Cytarabine, Granulocyte colony-stimulating factor, and Idarubicin with Gemtuzumab Ozogamicin improves event-free survival in younger patients with newly diagnosed AML and overall survival in patients with NPM1 and FLT3 mutations. J Clin Oncol 2024:JCO2300943. 10.1200/JCO.23.00943 PMC11003505 38215358

[23] Nikolaenko L, Nademanee A (2020). Brentuximab vedotin and its use in the treatment of advanced Hodgkin’s lymphoma. Future Oncol.

[24] Younes A, Bartlett NL, Leonard JP, Kennedy DA, Lynch CM, Sievers EL, Forero-Torres A (2010). Brentuximab vedotin (SGN-35) for relapsed CD30-positive lymphomas. N Engl J Med.

[25] Katz J, Janik JE, Younes A (2011). Brentuximab Vedotin (SGN-35). Clin Cancer Res.

[26] Horwitz SM, Scarisbrick JJ, Dummer R, Whittaker S, Duvic M, Kim YH, Quaglino P, Zinzani PL, Bechter O, Eradat H (2021). Randomized phase 3 ALCANZA study of brentuximab vedotin vs physician’s choice in cutaneous T-cell lymphoma: final data. Blood Adv.

[27] Richardson NC, Kasamon YL, Chen H, de Claro RA, Ye J, Blumenthal GM, Farrell AT, Pazdur R (2019). FDA approval Summary: Brentuximab Vedotin in First-Line treatment of Peripheral T-Cell Lymphoma. Oncologist.

[28] Zhang X, Qiao H, Chai X, Gao X, Ma R, Li Y, Zhu Z, Zhang M (2023). Brentuximab vedotin in treating Chinese patients with lymphoma: a multicenter, real-world study. Cancer Med.

[29] Rubinstein PG, Moore PC, Bimali M, Lee JY, Rudek MA, Chadburn A, Ratner L, Henry DH, Cesarman E, DeMarco CE (2023). Brentuximab vedotin with AVD for stage II-IV HIV-related Hodgkin lymphoma (AMC 085): phase 2 results from an open-label, single arm, multicentre phase 1/2 trial. Lancet Haematol.

[30] Ballantyne A, Dhillon S (2013). Trastuzumab emtansine: first global approval. Drugs.

[31] Verma S, Miles D, Gianni L, Krop IE, Welslau M, Baselga J, Pegram M, Oh DY, Diéras V, Guardino E (2012). Trastuzumab emtansine for HER2-positive advanced breast cancer. N Engl J Med.

[32] Hurvitz SA, Dirix L, Kocsis J, Bianchi GV, Lu J, Vinholes J, Guardino E, Song C, Tong B, Ng V (2013). Phase II randomized study of trastuzumab emtansine versus trastuzumab plus docetaxel in patients with human epidermal growth factor receptor 2-positive metastatic breast cancer. J Clin Oncol.

[33] Krop IE, Kim SB, Martin AG, LoRusso PM, Ferrero JM, Badovinac-Crnjevic T, Hoersch S, Smitt M, Wildiers H (2017). Trastuzumab emtansine versus treatment of physician’s choice in patients with previously treated HER2-positive metastatic breast cancer (TH3RESA): final overall survival results from a randomised open-label phase 3 trial. Lancet Oncol.

[34] Sanglier T, Fabi A, Flores C, Flahavan E, Lindegger N, Montemurro F (2019). Use of trastuzumab emtansine (T-DM1; K) after pertuzumab + trastuzumab (PH) in patients with HER2-positive metastatic breast cancer (mBC): challenges in assessing effectiveness of treatment sequencing in the real world (RW). Ann Oncol.

[35] Gradishar WJ, Moran MS, Abraham J, Aft R, Agnese D, Allison KH, Anderson B, Burstein HJ, Chew H, Dang C (2022). Breast Cancer, Version 3.2022, NCCN Clinical Practice guidelines in Oncology. J Natl Compr Canc Netw.

[36] von Minckwitz G, Huang CS, Mano MS, Loibl S, Mamounas EP, Untch M, Wolmark N, Rastogi P, Schneeweiss A, Redondo A (2019). Trastuzumab Emtansine for residual invasive HER2-Positive breast Cancer. N Engl J Med.

[37] Tolaney SM, Tayob N, Dang C, Yardley DA, Isakoff SJ, Valero V, Faggen M, Mulvey T, Bose R, Hu J (2021). Adjuvant trastuzumab Emtansine Versus Paclitaxel in Combination with Trastuzumab for Stage I HER2-Positive breast Cancer (ATEMPT): a Randomized Clinical Trial. J Clin Oncol.

[38] Harbeck N, Nitz UA, Christgen M, Kummel S, Braun M, Schumacher C, Potenberg J, Tio J, Aktas B, Forstbauer H (2023). De-escalated Neoadjuvant Trastuzumab-Emtansine with or without endocrine therapy Versus Trastuzumab with Endocrine Therapy in HR+/HER2 + early breast Cancer: 5-Year survival in the WSG-ADAPT-TP Trial. J Clin Oncol.

[39] Jenkins S, Zhang W, Steinberg SM, Nousome D, Houston N, Wu X, Armstrong TS, Burton E, Smart DD, Shah R (2023). Phase I study and cell-free DNA analysis of T-DM1 and metronomic temozolomide for secondary Prevention of HER2-Positive breast Cancer Brain metastases. Clin Cancer Res.

[40] de Vries EGE, Ruschoff J, Lolkema M, Tabernero J, Gianni L, Voest E, de Groot DJA, Castellano D, Erb G, Naab J (2023). Phase II study (KAMELEON) of single-agent T-DM1 in patients with HER2-positive advanced urothelial bladder cancer or pancreatic cancer/cholangiocarcinoma. Cancer Med.

[41] Cesano A, Gayko U (2003). CD22 as a target of passive immunotherapy. Semin Oncol.

[42] Lamb YN (2017). Inotuzumab Ozogamicin: First Global approval. Drugs.

[43] Thomas X. Profile of inotuzumab ozogamicin and its potential in the treatment of acute lymphoblastic leukemia. Blood Lymphatic Cancer: Targets Therapy 2014, 2014:1.

[44] Thomas X (2012). Inotuzumab ozogamicin in the treatment of B-cell acute lymphoblastic leukemia. Expert Opin Investig Drugs.

[45] Kantarjian HM, DeAngelo DJ, Stelljes M, Martinelli G, Liedtke M, Stock W, Gökbuget N, O’Brien S, Wang K, Wang T (2016). Inotuzumab Ozogamicin versus Standard Therapy for Acute Lymphoblastic Leukemia. N Engl J Med.

[46] Kantarjian HM, Su Y, Jabbour EJ, Bhattacharyya H, Yan E, Cappelleri JC, Marks DI (2018). Patient-reported outcomes from a phase 3 randomized controlled trial of inotuzumab ozogamicin versus standard therapy for relapsed/refractory acute lymphoblastic leukemia. Cancer.

[47] Kantarjian HM, DeAngelo DJ, Stelljes M, Liedtke M, Stock W, Gökbuget N, O’Brien SM, Jabbour E, Wang T, Liang White J (2019). Inotuzumab ozogamicin versus standard of care in relapsed or refractory acute lymphoblastic leukemia: final report and long-term survival follow-up from the randomized, phase 3 INO-VATE study. Cancer.

[48] DeAngelo DJ, Stock W, Stein AS, Shustov A, Liedtke M, Schiffer CA, Vandendries E, Liau K, Ananthakrishnan R, Boni J (2017). Inotuzumab ozogamicin in adults with relapsed or refractory CD22-positive acute lymphoblastic leukemia: a phase 1/2 study. Blood Adv.

[49] Pennesi E, Michels N, Brivio E, van der Velden VHJ, Jiang Y, Thano A, Ammerlaan AJC, Boer JM, Beverloo HB, Sleight B (2022). Inotuzumab ozogamicin as single agent in pediatric patients with relapsed and refractory acute lymphoblastic leukemia: results from a phase II trial. Leukemia.

[50] Metheny L, Sobecks RM, Cho C, Fu P, Margevicius S, Wang J, Ciarrone L, Kopp S, Convents R, Majhail NS et al. A multicenter study of posttransplant low-dose inotuzumab ozogamicin to prevent relapse of acute lymphoblastic leukemia. Blood Adv 2024. 10.1182/bloodadvances.2023011514 PMC10945150 38170741

[51] Jabbour E, Haddad FG, Short NJ, Senapati J, Jain N, Sasaki K, Jorgensen J, Wang SA, Alvarado Y, Wang X (2024). Phase 2 study of inotuzumab ozogamicin for measurable residual disease in acute lymphoblastic leukemia in remission. Blood.

[52] Dhillon S (2018). Moxetumomab Pasudotox: First Global approval. Drugs.

[53] Kreitman RJ, Dearden C, Zinzani PL, Delgado J, Karlin L, Robak T, et al. Moxetumomab pasudotox in relapsed/refractory hairy cell leukemia. Leukemia. 2018;32:1768–77. 10.1038/s41375-018-0210-1 PMC6087717 30030507

[54] Kreitman RJ, Dearden C, Zinzani PL, Delgado J, Robak T, le Coutre PD, Gjertsen BT, Troussard X, Roboz GJ, Karlin L (2021). Moxetumomab pasudotox in heavily pre-treated patients with relapsed/refractory hairy cell leukemia (HCL): long-term follow-up from the pivotal trial. J Hematol Oncol.

[55] Deeks ED (2019). Polatuzumab Vedotin: First Global approval. Drugs.

[56] Sehn LH, Herrera AF, Flowers CR, Kamdar MK, McMillan A, Hertzberg M, Assouline S, Kim TM, Kim WS, Ozcan M (2020). Polatuzumab Vedotin in relapsed or refractory diffuse large B-Cell lymphoma. J Clin Oncol.

[57] Tilly H, Morschhauser F, Sehn LH, Friedberg JW, Trněný M, Sharman JP, Herbaux C, Burke JM, Matasar M, Rai S (2022). Polatuzumab Vedotin in previously untreated diffuse large B-Cell lymphoma. N Engl J Med.

[58] Song Y, Tilly H, Rai S, Zhang H, Jin J, Goto H, Terui Y, Shin HJ, Kim WS, Cao J (2023). Polatuzumab vedotin in previously untreated DLBCL: an Asia subpopulation analysis from the phase 3 POLARIX trial. Blood.

[59] Lasater EA, Amin DN, Bannerji R, Mali RS, Barrett K, Rys RN, Oeh J, Lin E, Sterne-Weiler T, Ingalla ER (2023). Targeting MCL-1 and BCL-2 with polatuzumab vedotin and venetoclax overcomes treatment resistance in R/R non-hodgkin lymphoma: results from preclinical models and a phase ib study. Am J Hematol.

[60] Budde LE, Olszewski AJ, Assouline S, Lossos IS, Diefenbach C, Kamdar M, Ghosh N, Modi D, Sabry W, Naik S (2024). Mosunetuzumab with polatuzumab vedotin in relapsed or refractory aggressive large B cell lymphoma: a phase 1b/2 trial. Nat Med.

[61] Abrisqueta P, Gonzalez-Barca E, Panizo C, Perez JMA, Miall F, Bastos-Oreiro M, Triguero A, Banerjee L, McMillan A, Seymour E (2024). Polatuzumab vedotin plus rituximab and lenalidomide in patients with relapsed or refractory diffuse large B-cell lymphoma: a cohort of a multicentre, single-arm, phase 1b/2 study. Lancet Haematol.

[62] Heath EI, Rosenberg JE (2021). The biology and rationale of targeting nectin-4 in urothelial carcinoma. Nat Rev Urol.

[63] Wong JL, Rosenberg JE (2021). Targeting nectin-4 by antibody-drug conjugates for the treatment of urothelial carcinoma. Expert Opin Biol Ther.

[64] Hanna KS (2020). Enfortumab vedotin to treat urothelial carcinoma. Drugs Today (Barc).

[65] Rosenberg J, Sridhar SS, Zhang J, Smith D, Ruether D, Flaig TW, Baranda J, Lang J, Plimack ER, Sangha R (2020). EV-101: a phase I study of single-Agent Enfortumab Vedotin in patients with nectin-4-Positive solid tumors, including metastatic urothelial carcinoma. J Clin Oncol.

[66] McGregor B, O’Donnell PH, Balar A, Petrylak D, Rosenberg J, Yu EY, Quinn DI, Heath EI, Campbell M, Hepp Z (2022). Health-related quality of life of patients with locally Advanced or Metastatic Urothelial Cancer treated with Enfortumab Vedotin after platinum and PD-1/PD-L1 inhibitor therapy: results from cohort 1 of the phase 2 EV-201 clinical trial. Eur Urol.

[67] Rosenberg JE, Powles T, Sonpavde GP, Loriot Y, Duran I, Lee JL, Matsubara N, Vulsteke C, Castellano D, Mamtani R (2023). EV-301 long-term outcomes: 24-month findings from the phase III trial of enfortumab vedotin versus chemotherapy in patients with previously treated advanced urothelial carcinoma. Ann Oncol.

[68] Koshkin VS, Henderson N, James M, Natesan D, Freeman D, Nizam A, Su CT, Khaki AR, Osterman CK, Glover MJ (2022). Efficacy of enfortumab vedotin in advanced urothelial cancer: analysis from the Urothelial Cancer Network to investigate therapeutic experiences (UNITE) study. Cancer.

[69] Hoimes CJ, Flaig TW, Milowsky MI, Friedlander TW, Bilen MA, Gupta S, Srinivas S, Merchan JR, McKay RR, Petrylak DP (2023). Enfortumab Vedotin Plus Pembrolizumab in previously untreated Advanced Urothelial Cancer. J Clin Oncol.

[70] Milowsky MI, O’Donnell PH, Hoimes CJ, Petrylak DP, Flaig TW, Moon HH, Friedlander TW, Mar N, McKay RR, Srinivas S et al. Patient-reported outcomes in patients with Advanced Urothelial Cancer who are Ineligible for Cisplatin and treated with First-Line Enfortumab Vedotin alone or with Pembrolizumab. J Clin Oncol 2024:JCO2301547. 10.1200/JCO.23.01547 PMC11095879 38215355

[71] O’Donnell PH, Milowsky MI, Petrylak DP, Hoimes CJ, Flaig TW, Mar N, Moon HH, Friedlander TW, McKay RR, Bilen MA (2023). Enfortumab Vedotin with or without Pembrolizumab in Cisplatin-Ineligible patients with previously untreated locally Advanced or Metastatic Urothelial Cancer. J Clin Oncol.

[72] Ogitani Y, Hagihara K, Oitate M, Naito H, Agatsuma T (2016). Bystander killing effect of DS-8201a, a novel anti-human epidermal growth factor receptor 2 antibody-drug conjugate, in tumors with human epidermal growth factor receptor 2 heterogeneity. Cancer Sci.

[73] Ogitani Y, Aida T, Hagihara K, Yamaguchi J, Ishii C, Harada N, Soma M, Okamoto H, Oitate M, Arakawa S (2016). DS-8201a, A Novel HER2-Targeting ADC with a novel DNA topoisomerase I inhibitor, demonstrates a Promising Antitumor Efficacy with differentiation from T-DM1. Clin Cancer Res.

[74] Keam SJ (2020). Trastuzumab Deruxtecan: first approval. Drugs.

[75] Tamura K, Tsurutani J, Takahashi S, Iwata H, Krop IE, Redfern C, Sagara Y, Doi T, Park H, Murthy RK (2019). Trastuzumab Deruxtecan (DS-8201a) in patients with advanced HER2-positive breast cancer previously treated with trastuzumab emtansine: a dose-expansion, phase 1 study. Lancet Oncol.

[76] Modi S, Saura C, Yamashita T, Park YH, Kim SB, Tamura K, Andre F, Iwata H, Ito Y, Tsurutani J (2020). Trastuzumab Deruxtecan in previously treated HER2-Positive breast Cancer. N Engl J Med.

[77] Jerusalem G, Park YH, Yamashita T, Hurvitz SA, Modi S, Andre F, Krop IE, Gonzalez Farre X, You B, Saura C (2022). Trastuzumab Deruxtecan in HER2-Positive metastatic breast Cancer patients with brain metastases: a DESTINY-Breast01 subgroup analysis. Cancer Discov.

[78] Saura C, Modi S, Krop I, Park YH, Kim SB, Tamura K, Iwata H, Tsurutani J, Sohn J, Mathias E et al. Trastuzumab Deruxtecan in previously treated patients with HER2-positive metastatic breast cancer: updated survival results from a phase II trial (DESTINY-Breast01). Ann Oncol 2023. 10.1016/j.annonc.2023.12.001 PMC11322859 38092229

[79] Bartsch R, Berghoff AS, Furtner J, Marhold M, Bergen ES, Roider-Schur S, Starzer AM, Forstner H, Rottenmanner B, Dieckmann K (2022). Trastuzumab Deruxtecan in HER2-positive breast cancer with brain metastases: a single-arm, phase 2 trial. Nat Med.

[80] Andre F, Hee Park Y, Kim SB, Takano T, Im SA, Borges G, Lima JP, Aksoy S, Gavila Gregori J, De Laurentiis M (2023). Trastuzumab Deruxtecan versus treatment of physician’s choice in patients with HER2-positive metastatic breast cancer (DESTINY-Breast02): a randomised, open-label, multicentre, phase 3 trial. Lancet.

[81] Hurvitz SA, Hegg R, Chung WP, Im SA, Jacot W, Ganju V, Chiu JWY, Xu B, Hamilton E, Madhusudan S (2023). Trastuzumab deruxtecan versus trastuzumab emtansine in patients with HER2-positive metastatic breast cancer: updated results from DESTINY-Breast03, a randomised, open-label, phase 3 trial. Lancet.

[82] Curigliano G, Dunton K, Rosenlund M, Janek M, Cathcart J, Liu Y, Fasching PA, Iwata H (2023). Patient-reported outcomes and hospitalization data in patients with HER2-positive metastatic breast cancer receiving trastuzumab deruxtecan or trastuzumab emtansine in the phase III DESTINY-Breast03 study. Ann Oncol.

[83] Modi S, Jacot W, Yamashita T, Sohn J, Vidal M, Tokunaga E, Tsurutani J, Ueno NT, Prat A, Chae YS (2022). Trastuzumab Deruxtecan in previously treated HER2-Low advanced breast Cancer. N Engl J Med.

[84] Narayan P, Dilawari A, Osgood C, Feng Z, Bloomquist E, Pierce WF, Jafri S, Kalavar S, Kondratovich M, Jha P (2023). US Food and Drug Administration approval Summary: fam-trastuzumab deruxtecan-nxki for human epidermal growth factor receptor 2-Low unresectable or metastatic breast Cancer. J Clin Oncol.

[85] Zaman S, Jadid H, Denson AC, Gray JE (2019). Targeting Trop-2 in solid tumors: future prospects. Onco Targets Ther.

[86] Vranic S, Gatalica Z (2022). Trop-2 protein as a therapeutic target: a focused review on Trop-2-based antibody-drug conjugates and their predictive biomarkers. Bosn J Basic Med Sci.

[87] Syed YY (2020). Sacituzumab Govitecan: first approval. Drugs.

[88] Starodub AN, Ocean AJ, Shah MA, Guarino MJ, Picozzi VJ, Vahdat LT, Thomas SS, Govindan SV, Maliakal PP, Wegener WA (2015). First-in-human trial of a Novel anti-trop-2 Antibody-SN-38 Conjugate, Sacituzumab Govitecan, for the treatment of diverse metastatic solid tumors. Clin Cancer Res.

[89] Bardia A, Mayer IA, Vahdat LT, Tolaney SM, Isakoff SJ, Diamond JR, O’Shaughnessy J, Moroose RL, Santin AD, Abramson VG (2019). Sacituzumab Govitecan-Hziy in Refractory Metastatic Triple-negative breast Cancer. N Engl J Med.

[90] Rugo HS, Bardia A, Marme F, Cortes J, Schmid P, Loirat D, Tredan O, Ciruelos E, Dalenc F, Gomez Pardo P (2023). Overall survival with sacituzumab govitecan in hormone receptor-positive and human epidermal growth factor receptor 2-negative metastatic breast cancer (TROPiCS-02): a randomised, open-label, multicentre, phase 3 trial. Lancet.

[91] Spring LM, Tolaney SM, Fell G, Bossuyt V, Abelman RO, Wu B, Maheswaran S, Trippa L, Comander A, Mulvey T et al. Response-guided neoadjuvant sacituzumab govitecan for localized triple-negative breast cancer: results from the NeoSTAR trial. Ann Oncol 2023. 10.1016/j.annonc.2023.11.018 38092228

[92] Tagawa ST, Faltas BM, Lam ET, Saylor PJ, Bardia A, Hajdenberg J, Morgans AK, Lim EA, Kalinsky K, Simpson PS (2019). Sacituzumab govitecan (IMMU-132) in patients with previously treated metastatic urothelial cancer (mUC): results from a phase I/II study. J Clin Oncol.

[93] Tagawa ST, Balar AV, Petrylak DP, Kalebasty AR, Loriot Y, Fléchon A, Jain RK, Agarwal N, Bupathi M, Barthelemy P (2021). TROPHY-U-01: a phase II open-label study of Sacituzumab Govitecan in patients with metastatic urothelial carcinoma progressing after platinum-based chemotherapy and checkpoint inhibitors. J Clin Oncol.

[94] Loriot Y, Petrylak DP, Kalebasty AR, Flechon A, Jain RK, Gupta S, Bupathi M, Beuzeboc P, Palmbos P, Balar AV et al. TROPHY-U-01, a phase II open-label study of sacituzumab govitecan in patients with metastatic urothelial carcinoma progressing after platinum-based chemotherapy and checkpoint inhibitors: updated safety and efficacy outcomes. Ann Oncol 2024. 10.1016/j.annonc.2024.01.002 38244927

[95] Chou J, Trepka K, Sjöström M, Egusa EA, Chu CE, Zhu J, Chan E, Gibb EA, Badura ML, Contreras-Sanz A (2022). TROP2 Expression across Molecular Subtypes of Urothelial Carcinoma and Enfortumab Vedotin-resistant cells. Eur Urol Oncol.

[96] McGregor BA, Sonpavde GP, Kwak L, Regan MM, Gao X, Hvidsten H, Mantia CM, Wei XX, Berchuck JE, Berg SA (2024). The double antibody drug Conjugate (DAD) phase I trial: sacituzumab govitecan plus enfortumab vedotin for metastatic urothelial carcinoma. Ann Oncol.

[97] Jiang J, Li S, Shan X, Wang L, Ma J, Huang M, Dong L, Chen F (2020). Preclinical safety profile of disitamab vedotin: a novel anti-HER2 antibody conjugated with MMAE. Toxicol Lett.

[98] Deeks ED (2021). Disitamab Vedotin: first approval. Drugs.

[99] Xu Y, Wang Y, Gong J, Zhang X, Peng Z, Sheng X, Mao C, Fan Q, Bai Y, Ba Y (2021). Phase I study of the recombinant humanized anti-HER2 monoclonal antibody-MMAE conjugate RC48-ADC in patients with HER2-positive advanced solid tumors. Gastric Cancer.

[100] Peng Z, Liu T, Wei J, Wang A, He Y, Yang L, Zhang X, Fan N, Luo S, Li Z (2021). Efficacy and safety of a novel anti-HER2 therapeutic antibody RC48 in patients with HER2-overexpressing, locally advanced or metastatic gastric or gastroesophageal junction cancer: a single-arm phase II study. Cancer Commun (Lond).

[101] Nie C, Xu W, Guo Y, Gao X, Lv H, Chen B, Wang J, Liu Y, Zhao J, Wang S (2023). Immune checkpoint inhibitors enhanced the antitumor efficacy of disitamab vedotin for patients with HER2-positive or HER2-low advanced or metastatic gastric cancer: a multicenter real-world study. BMC Cancer.

[102] Alameddine R, Mallea P, Shahab F, Zakharia Y (2023). Antibody drug conjugates in bladder Cancer: current milestones and future perspectives. Curr Treat Options Oncol.

[103] Yu J, Wu S, Li R, Jiang Y, Zheng J, Li Z, Li M, Xin K, Guan X, Li S, Chen X. Novel ADCs and combination therapy in urothelial carcinoma: latest updates from the 2023 ASCO-GU Cancers Symposium. *J Hematol Oncol* 2023, 16:85. 10.1186/s13045-023-01475-9 PMC10385919 37507780

[104] Sheng X, Yan X, Wang L, Shi Y, Yao X, Luo H, Shi B, Liu J, He Z, Yu G (2021). Open-label, Multicenter, Phase II study of RC48-ADC, a HER2-Targeting antibody-drug Conjugate, in patients with locally Advanced or Metastatic Urothelial Carcinoma. Clin Cancer Res.

[105] Sheng X, Wang L, He Z, Shi Y, Luo H, Han W, et al. Efficacy and safety of disitamab vedotin in patients with human epidermal growth factor receptor 2-positive locally advanced or metastatic urothelial carcinoma: A combined analysis of two phase II clinical trials. J Clin Oncol. 2023:JCO2202912. 10.1200/JCO.22.02912 PMC11095880 37988648

[106] Chen M, Yao K, Cao M, Liu H, Xue C, Qin T, Meng L, Zheng Z, Qin Z, Zhou F (2023). HER2-targeting antibody-drug conjugate RC48 alone or in combination with immunotherapy for locally advanced or metastatic urothelial carcinoma: a multicenter, real-world study. Cancer Immunol Immunother.

[107] Wei Y, Zhang R, Yu C, Hong Z, Lin L, Li T, Chen J (2023). Disitamab vedotin in combination with immune checkpoint inhibitors for locally and locally advanced bladder urothelial carcinoma: a two-center’s real-world study. Front Pharmacol.

[108] Xu J, Zhang H, Zhang L, Chu X, Li Y, Li G, Nie C, Wang M, Guo Y (2023). Real-world effectiveness and safety of RC48-ADC alone or in combination with PD-1 inhibitors for patients with locally advanced or metastatic urothelial carcinoma: a multicenter, retrospective clinical study. Cancer Med.

[109] Wen F, Lin T, Zhang P, Shen Y (2023). RC48-ADC combined with tislelizumab as neoadjuvant treatment in patients with HER2-positive locally advanced muscle-invasive urothelial bladder cancer: a multi-center phase Ib/II study (HOPE-03). Front Oncol.

[110] Lee A (2021). Loncastuximab Tesirine: first approval. Drugs.

[111] Hamadani M, Radford J, Carlo-Stella C, Caimi PF, Reid E, O’Connor OA, Feingold JM, Ardeshna KM, Townsend W, Solh M (2021). Final results of a phase 1 study of loncastuximab tesirine in relapsed/refractory B-cell non-hodgkin lymphoma. Blood.

[112] Caimi PF, Ai WZ, Alderuccio JP, Ardeshna KM, Hamadani M, Hess B, Kahl BS, Radford J, Solh M, Stathis A et al. Loncastuximab tesirine in relapsed/refractory diffuse large B-cell lymphoma: long-term efficacy and safety from the phase 2 LOTIS-2 study. Haematologica 2023. 10.3324/haematol.2023.283459 PMC10985439 37646659

[113] Caimi PF, Ai W, Alderuccio JP, Ardeshna KM, Hamadani M, Hess B, Kahl BS, Radford J, Solh M, Stathis A (2021). Loncastuximab tesirine in relapsed or refractory diffuse large B-cell lymphoma (LOTIS-2): a multicentre, open-label, single-arm, phase 2 trial. Lancet Oncol.

[114] Hamadani M, Spira A, Zhou X, Liao L, Chen L, Radford J, Ai W, Solh M, Ardeshna KM, Hess B (2024). Clinical outcomes of older and younger patients treated with loncastuximab tesirine in the LOTIS-2 clinical trial. Blood Adv.

[115] Förster Y, Meye A, Albrecht S, Schwenzer B (2006). Tissue factor and tumor: clinical and laboratory aspects. Clin Chim Acta.

[116] van den Berg YW, Osanto S, Reitsma PH, Versteeg HH (2012). The relationship between tissue factor and cancer progression: insights from bench and bedside. Blood.

[117] Markham A (2021). Tisotumab Vedotin: first approval. Drugs.

[118] de Bono JS, Concin N, Hong DS, Thistlethwaite FC, Machiels JP, Arkenau HT, Plummer R, Jones RH, Nielsen D, Windfeld K (2019). Tisotumab vedotin in patients with advanced or metastatic solid tumours (InnovaTV 201): a first-in-human, multicentre, phase 1–2 trial. Lancet Oncol.

[119] Coleman RL, Lorusso D, Gennigens C, González-Martín A, Randall L, Cibula D, Lund B, Woelber L, Pignata S, Forget F (2021). Efficacy and safety of tisotumab vedotin in previously treated recurrent or metastatic cervical cancer (innovaTV 204/GOG-3023/ENGOT-cx6): a multicentre, open-label, single-arm, phase 2 study. Lancet Oncol.

[120] Yonemori K, Kuboki Y, Hasegawa K, Iwata T, Kato H, Takehara K, Hirashima Y, Kato H, Passey C, Buchbjerg JK (2022). Tisotumab vedotin in Japanese patients with recurrent/metastatic cervical cancer: results from the innovaTV 206 study. Cancer Sci.

[121] Vergote I, Van Nieuwenhuysen E, O’Cearbhaill RE, Westermann A, Lorusso D, Ghamande S, Collins DC, Banerjee S, Mathews CA, Gennigens C (2023). Tisotumab Vedotin in Combination with Carboplatin, Pembrolizumab, or Bevacizumab in recurrent or metastatic cervical Cancer: results from the innovaTV 205/GOG-3024/ENGOT-cx8 study. J Clin Oncol.

[122] Salazar MD, Ratnam M (2007). The folate receptor: what does it promise in tissue-targeted therapeutics?. Cancer Metastasis Rev.

[123] Ledermann JA, Canevari S, Thigpen T (2015). Targeting the folate receptor: diagnostic and therapeutic approaches to personalize cancer treatments. Ann Oncol.

[124] Parker N, Turk MJ, Westrick E, Lewis JD, Low PS, Leamon CP (2005). Folate receptor expression in carcinomas and normal tissues determined by a quantitative radioligand binding assay. Anal Biochem.

[125] O’Shannessy DJ, Yu G, Smale R, Fu YS, Singhal S, Thiel RP, Somers EB, Vachani A (2012). Folate receptor alpha expression in lung cancer: diagnostic and prognostic significance. Oncotarget.

[126] O’Shannessy DJ, Somers EB, Maltzman J, Smale R, Fu YS (2012). Folate receptor alpha (FRA) expression in breast cancer: identification of a new molecular subtype and association with triple negative disease. Springerplus.

[127] Heo YA (2023). Mirvetuximab Soravtansine: first approval. Drugs.

[128] Dilawari A, Shah M, Ison G, Gittleman H, Fiero MH, Shah A, et al. FDA approval Summary: Mirvetuximab Soravtansine-Gynx for FRɑ-Positive, platinum-resistant ovarian Cancer. Clin Cancer Res. 2023;29:3835–40. 10.1158/1078-0432.CCR-23-0991 PMC10592645 37212825

[129] Moore KN, Martin LP, O’Malley DM, Matulonis UA, Konner JA, Perez RP, Bauer TM, Ruiz-Soto R, Birrer MJ (2017). Safety and activity of Mirvetuximab Soravtansine (IMGN853), a folate receptor alpha-targeting antibody-drug Conjugate, in platinum-resistant ovarian, fallopian tube, or primary peritoneal Cancer: a phase I expansion study. J Clin Oncol.

[130] Martin LP, Konner JA, Moore KN, Seward SM, Matulonis UA, Perez RP, Su Y, Berkenblit A, Ruiz-Soto R, Birrer MJ (2017). Characterization of folate receptor alpha (FRα) expression in archival tumor and biopsy samples from relapsed epithelial ovarian cancer patients: a phase I expansion study of the FRα-targeting antibody-drug conjugate mirvetuximab soravtansine. Gynecol Oncol.

[131] Matulonis UA, Lorusso D, Oaknin A, Pignata S, Dean A, Denys H, et al. Efficacy and safety of Mirvetuximab Soravtansine in patients with platinum-resistant ovarian Cancer with high folate receptor alpha expression: results from the SORAYA study. J Clin Oncol. 2023;41:2436–45. 10.1200/JCO.22.01900 PMC10150846 36716407

[132] Moore KN, Oza AM, Colombo N, Oaknin A, Scambia G, Lorusso D, Konecny GE, Banerjee S, Murphy CG, Tanyi JL (2021). Phase III, randomized trial of mirvetuximab soravtansine versus chemotherapy in patients with platinum-resistant ovarian cancer: primary analysis of FORWARD I. Ann Oncol.

[133] Moore KN, Angelergues A, Konecny GE, Garcia Y, Banerjee S, Lorusso D, Lee JY, Moroney JW, Colombo N, Roszak A (2023). Mirvetuximab Soravtansine in FRalpha-Positive, platinum-resistant ovarian Cancer. N Engl J Med.

[134] Moore KN, O’Malley DM, Vergote I, Martin LP, Gonzalez-Martin A, Malek K, Birrer MJ (2018). Safety and activity findings from a phase 1b escalation study of mirvetuximab soravtansine, a folate receptor alpha (FRalpha)-targeting antibody-drug conjugate (ADC), in combination with carboplatin in patients with platinum-sensitive ovarian cancer. Gynecol Oncol.

[135] O’Malley DM, Matulonis UA, Birrer MJ, Castro CM, Gilbert L, Vergote I, Martin LP, Mantia-Smaldone GM, Martin AG, Bratos R (2020). Phase ib study of mirvetuximab soravtansine, a folate receptor alpha (FRalpha)-targeting antibody-drug conjugate (ADC), in combination with bevacizumab in patients with platinum-resistant ovarian cancer. Gynecol Oncol.

[136] Gilbert L, Oaknin A, Matulonis UA, Mantia-Smaldone GM, Lim PC, Castro CM, Provencher D, Memarzadeh S, Method M, Wang J (2023). Safety and efficacy of mirvetuximab soravtansine, a folate receptor alpha (FRalpha)-targeting antibody-drug conjugate (ADC), in combination with bevacizumab in patients with platinum-resistant ovarian cancer. Gynecol Oncol.

[137] Dumontet C, Reichert JM, Senter PD, Lambert JM, Beck A (2023). Antibody-drug conjugates come of age in oncology. Nat Rev Drug Discov.

[138] Nicolo E, Giugliano F, Ascione L, Tarantino P, Corti C, Tolaney SM, Cristofanilli M, Curigliano G. Combining antibody-drug conjugates with immunotherapy in solid tumors: current landscape and future perspectives. Cancer Treat Rev. 2022;106:102395. 10.1016/j.ctrv.2022.102395 35468539

[139] Thurber GM, Schmidt MM, Wittrup KD (2008). Antibody tumor penetration: transport opposed by systemic and antigen-mediated clearance. Adv Drug Deliv Rev.

[140] Mullard A (2013). Maturing antibody-drug conjugate pipeline hits 30. Nat Rev Drug Discov.

[141] Beck A, Goetsch L, Dumontet C, Corvaïa N (2017). Strategies and challenges for the next generation of antibody-drug conjugates. Nat Rev Drug Discov.

[142] Walker JA, Sorkin MR, Ledesma F, Kabaria SR, Barfield RM, Rabuka D, Alabi CA. Hydrophilic sequence-defined cross-linkers for antibody–drug conjugates. Bioconjug Chem. 2019;30:2982–88. 10.1021/acs.bioconjchem.9b00713 31671265

[143] Jin Y, Schladetsch MA, Huang X, Balunas MJ, Wiemer AJ. Stepping forward in antibody-drug conjugate development. Pharmacol Ther. 2022;229:107917. 10.1016/j.pharmthera.2021.107917 PMC8702582 34171334

[144] Schumacher D, Hackenberger CP, Leonhardt H, Helma J. Current status: site-specific antibody drug conjugates. J Clin Immunol. 2016;36(Suppl 1):100–7. 10.1007/s10875-016-0265-6 PMC4891387 27003914

[145] Perez HL, Cardarelli PM, Deshpande S, Gangwar S, Schroeder GM, Vite GD, Borzilleri RM. Antibody-drug conjugates: current status and future directions. Drug Discov Today. 2014;19:869–81. 10.1016/j.drudis.2013.11.004 24239727

